# Visual Recovery after Primary Retinal Detachment Surgery: Biofeedback Rehabilitative Strategy

**DOI:** 10.1155/2016/8092396

**Published:** 2016-02-21

**Authors:** Enzo M. Vingolo, Serena Fragiotta, Daniela Domanico, Paolo G. Limoli, Marcella Nebbioso, Leopoldo Spadea

**Affiliations:** ^1^UOC Ophthalmology, Ospedale A. Fiorini Terracina, Sapienza University of Rome, 04120 Terracina, Italy; ^2^Centro Studi Ipovisione, Milano, Italy; ^3^Department of Ophthalmology, University of Rome, Rome, Italy

## Abstract

*Purpose*. To evaluate possible speeding up recovery time after retinal detachment (RD) surgery using biofeedback strategy.* Methods*. A total of 52 eyes were selected. After surgery, patients were divided into two groups: group A, including patients submitted to biofeedback with MP-1 strategy; group B, patients who received common care strategy. Biofeedback strategy was started 15 days after the suspension of cycloplegic eye drops in buckling procedure or after silicone oil removal in the vitrectomized eyes. Controls were scheduled at baseline and 6, 12, and 18 weeks.* Results*. At baseline, there was no significant difference in BCVA between groups (*P* = 0.4230). At the end of biofeedback treatment (WK 6) BCVA of group A was significantly better (*P* < 0.001) than group B and BCVA was still better in group A than group B at WK 12 (*P* = 0.028) and at WK 18 (*P* = 0.041).* Conclusions*. Visual recovery after RD surgery is still unclear, and it does not depend on entity of the RD. Our data demonstrate that in biofeedback group there was a significant recovery in visual performances that still remains evident after 3 months from the baseline.

## 1. Introduction

Visual recovery after surgery for macula-off retinal detachment (RD) is often discouraging because performances are very limited even if retinal reattachment is achieved. Successful reattachment of the macula after RD is often associated with incomplete visual recovery. Preoperative factors influencing macula recovery include preoperative visual acuity, duration, and height of detachment, and the presence of vitreomacular traction. Postoperative clinical findings associated with incomplete recovery include cystoid macular edema, epiretinal membranes, retinal folds, retinal pigment epithelium (RPE) migration, and persistent subretinal fluid (SRF) [1].

In this study we have evaluated the use of biofeedback rehabilitation with the MP-1 microperimeter (NIDEK Technologies Srl, Padova, Italy) as a possible strategy to speed up recovery time in operated eyes.

Fundus-related microperimetry (MP) is a functional measure of macular sensitivity. The MP-1 microperimeter measures several points in the patient's central field and effectively maps out microscotomas. An infrared camera establishes and tracks the patient's fixation, and the resulting visual field is registered onto the corresponding fundus photograph. Therefore, the functional defect can be localized anatomically onto the macular abnormality. Previous studies have shown that the MP-1 results are reproducible and comparable to standard automated perimetry [2, 3].

Visual rehabilitation is a therapeutic approach that has been applied to different ocular diseases characterized by visual deterioration and loss of stable central fixation [4]. The MP-1 microperimeter biofeedback examination allows the ophthalmologist to train the patient to fixate the target with a new preferred retinal locus (PRL), which can be defined as a discrete retinal area that contains more than 20% of the fixation points. The term “preferred retinal locus” (PRL) describes a retinal area that acts as a pseudofovea for visual tasks when a central macular scotoma affects visual performance. Moreover, a sizeable proportion of patients use more than one PRL for a given task. It has been also found that some patients exhibit a rereferencing of the oculomotor system to the PRL, which leads them to say that they are looking straight ahead when they are fixating with the PRL (i.e., when the eye is not in the primary position). This phenomenon has been referred to as adaptive eccentric fixation or oculomotor rereferencing [5].

## 2. Methods 

Fifty-two eyes of 52 patients (23 female and 29 male) were enrolled at the Department of Ophthalmology, “S.M. Goretti” Hospital between 2008 and early 2013. They suffered from RD treated with scleral buckle surgery or pars plana vitrectomy with silicon oil tamponade. The mean age was 58.24 ± 14.05 years (range: 27–88 years old).

The diagnosis of retinal detachment was based on a complete eye examination including best-corrected visual acuity (BCVA) test using a standard Snellen chart, slit-lamp biomicroscopy, intraocular pressure test, and binocular indirect ophthalmoscopy. We included only patients who accepted to perform visual rehabilitation treatment after an appropriate discussion about limitations, benefits, and risks of the procedure. Exclusion criteria were eyes undergoing reoperation of primary failure or redetachment, the presence of any other macular pathology such as macular hole, age-related macular degeneration, or macular oedema, patients with advanced glaucoma, and diabetic retinopathy. We also excluded patients with cognitive impairment, earing loss, or any other reason that hindered the proper execution of MP-1 biofeedback. Institutional Review Board (IRB) approval was obtained. All procedures adhered to the tenets of the Declaration of Helsinki. Each participant gave informed consent prior to enrollment in the study.

Twenty-three eyes of twenty-three patients had scleral buckle surgery combined with cryopexy for macula-off RD by an individual surgeon (Enzo M. Vingolo). Drainage of subretinal fluid was performed in all eyes. All operations were uncomplicated. Routine postoperative corticosteroids, antibiotics, and cycloplegics were prescribed and tapered over the subsequent postoperative weeks. Twenty-seven eyes of twenty-seven patients were submitted to pars plana vitrectomy (PPV) with 23-gauge system associated with the internal filing with silicone oil (polydimethylsiloxane (PDMS) 1000).

After the surgery patients were randomly divided into two group: group A, which included 25 eyes, 12 buckled and 13 with PPV and PDMS tamponade submitted to rehabilitation protocol with biofeedback (BF) MP-1; group B (control group), composed of 27 eyes, 13 buckled and 14 with PPV and PDMS tamponade, who received common care. Foveal reattachment after RD surgery was evaluated using Optical Coherence Tomography (OCT) scans. Patients who underwent PPV with gas tamponade were excluded because of small sample size.

Visual rehabilitation started 15 days after the suspension of cycloplegic eye drops in buckling procedure or after silicone oil removal in PPV eyes. At baseline, microperimetry and fixation test were performed using a standard protocol. A red cross of 1-degree and 1 unit thickness (10 minarc) was used as a fixation target. If the patient is not able to see it, a 2-degree red cross was used as fixation target. MP was performed using white background illumination of 4 asb (1.27 cd/m^2^) and stimulus size Goldmann III, with a projection time of 200 ms. A 45-loci customized grid covering the central 12°, centered on the fovea, was selected as pattern. We used a 4-to-2-staircase strategy and the initial projecting sensitivity was fixed at 16 dB. “Pretest” option was selected and spherical error was manually typed into the window before starting the examination. At the end of visual rehabilitation training only a red cross of 1-degree was used for all patients.

The new PRL was chosen based on baseline microperimetry. The rehabilitation protocol consisted of 10 training sessions of 10 minutes for each eye, performed once a week using the MP-1 acoustic target biofeedback examination. The patients were trained to fix the new PRL according to an audio feedback which advised them whether they were getting closer to the desired final fixation position. All the procedures were followed on a monitor.

The microperimetry was performed at baseline and at the end of visual rehabilitation protocol (i.e., after 10 weeks). We collected data about retinal sensitivity and fixation stability. Fixation stability was reported as bivariate contour ellipse area (BCEA, deg^2^). The BCEA was normalized by logarithmic transformation for statistical analysis (Shapiro-Wilk test, *P* < 0.05).

Best correct visual acuity (BCVA) was performed at baseline and 6, 12, and 18 weeks in both groups. BCVA was measured using a standard Snellen chart (CSO electronic chart, Firenze, Italy) and then converted to logarithm of the minimum angle of resolution (logMAR) for statistical analyses. Statistical analysis was performed using Student's *t*-test and *P* values less than 0.05 were considered statistically significant.

## 3. Results

The mean patient age was 58.24 ± 14.05 years (range: 27–88 years old). The patients submitted to biofeedback training with microperimetry MP-1 (group A, 25 eyes) had mean preoperative BCVA of 1.48 logMAR, and they underwent surgery after 2.3 days of diagnosis; group B (control group, 27 eyes) had mean preoperative BCVA of 1 logMAR, and they were submitted to surgery after 2.4 days of diagnosis. All participants completed the study protocol.

The data regarding retinal sensitivity and fixation stability at baseline and at the end of visual rehabilitation were summarized in Table 1.

**Figure 1:**
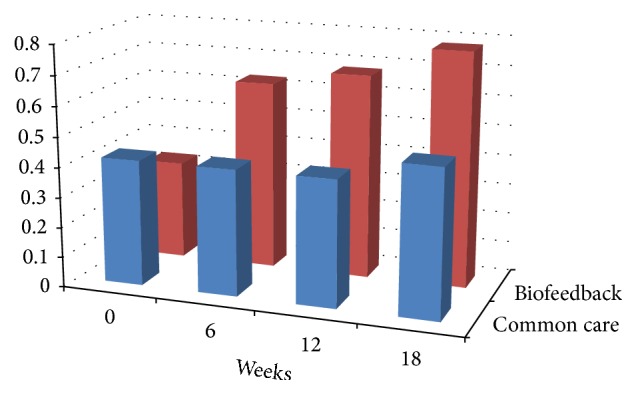
The graph shows BCVA of group A who performed rehabilitation with MP-1 biofeedback (BF) and BCVA of group B which was used as a control group at baseline and after 6 weeks, 12 weeks, and 18 weeks.

At baseline the mean BCVA was 0.6 ± 0.43 logMAR in group A and 0.66 ± 0.67 logMAR in group B with no statistical difference (*P* = 0.75). At 6 weeks after training with microperimetric biofeedback the mean BCVA of group A was significantly better than group B (0.27 ± 0.29 versus 0.67 ± 0.67 logMAR, *P* = 0.02). At 12 weeks the mean BCVA of group A was 0.18 ± 0.25 logMAR and 0.60 ± 0.66 logMAR in group B (*P* = 0.0109). At 18 weeks visual performances were still better in biofeedback group than in group B (0.15 ± 0.25 versus 0.58 ± 0.68 logMAR, *P* = 0.01) (Figure 1).

## 4. Discussion 

Scleral-buckling procedure is the most common surgical treatment of rhegmatogenous (primary) retinal detachment (RD), with or without intravitreal gas injection [6]. After scleral-buckling procedure, visual recovery is related to the preoperative and postoperative macular condition. A poor functional outcome is common because of postoperative complications, such as persistent subfoveal fluid, even in a preoperatively uninvolved macula [7], epiretinal membranes, and cystoids macular oedema [1, 8].

Pars plana vitrectomy (PPV) has become accepted as the treatment of choice for certain complicated retinal detachments. The most common indications are difficult retinal tears (e.g., giant or in macular region) [9, 10] or the presence of advanced proliferative vitreoretinopathy (PVR). In uncomplicated rhegmatogenous retinal detachment (RRD) external buckling procedures are usually preferred [11, 12].

Visual recovery after successful surgery for the macula-off rhegmatogenous retinal detachment is still debated. Salicone et al. studied the visual recovery after scleral-buckling procedure for retinal detachment in 672 patients, including 457 (68%) with macular detachment. They showed that the use of gas, drainage of subretinal fluid, and lens status did not influence final anatomic or visual results. Visual recovery after retinal reattachment was mostly influenced by macular involvement. Instead, the duration of macular detachment had surprisingly little influence on postoperative visual acuity. Macular detachment was the most important prognostic factor for anatomic and visual acuity success [13]. Kusaka et al. had retrospectively investigated the long-term visual recovery in 32 macula-off retinal detachments followed for more than 5 years after surgery. They found that the best-corrected visual acuities were better 5 years than 3 months by two lines or more in 17 eyes (53%), and it continued to improve for up to 10 years after surgery. The remaining 15 eyes demonstrated best-corrected acuities that remained within one line of the 3-month values. The eyes that demonstrated better postoperative long-term improvement were statistically correlated with younger age, no or mild myopia (less than −5.00 D), and shorter duration of macular detachment (30 days or less) [14].

In our study we have evaluated if it is possible to speed up recovery time in eyes after retinal detachment surgery with biofeedback rehabilitation with a MP-1 microperimeter. Biofeedback has been used for more than fifty years in rehabilitation to facilitate normal movement patterns after injury [15], ametropia, nystagmus, and amblyopia, in advanced glaucoma, and in different macular diseases such as age-related macular degeneration or myopia [16–28].

Our data demonstrate that in group A the biofeedback training allowed a significant recovery in visual acuity after clinical healing of the retina that still remains evident after 18 weeks. Moreover, the improvement of BCVA in the group who underwent biofeedback training was statistically significant being better than the control group. Finally, microperimetric functional parameters (e.g., retinal sensitivity and fixation stability) confirm a highly significant functional improvement of group A compared to controls.

Previous studies have demonstrated the efficacy of low-vision rehabilitation by means of MP-1 biofeedback examination with an improvement in visual acuity, fixation behavior, retinal sensitivity, and reading speed [4, 17].

Crossland et al. showed that the MP-1 uses cerebral plasticity and neurosensorial adaptation to the central scotoma of patients with macular diseases to improve their visual abilities and more manageable visual aids. Indeed, such patients often develop a new PRL, which can be defined as a discrete retinal area that contains more than 20% of the fixation points [5].

PRL was chosen according to microperimetry as an area of higher sensitivity and fixation points also indicated by colors of interpolated map or by numbers of the numerical map. The patients are asked to move their eyes according to an audio feedback, which indicates whether they are getting closer to PRL chosen by the ophthalmologist. Sound perception increases the conscious attention of the patient, thereby facilitating the lock-in of the visual target and increasing the permanence time of the fixation target itself on the retina. This mechanism facilitates stimuli transmission between intraretinal neurons as well as between the retina and brain, where the highest degree of stimuli processing takes place, thereby supporting a “remapping phenomenon” [18, 19]. The reasons of functional improvement obtained by BF training are due to the fact that we trained a “retinal motor” PRL, with appropriate retinal sensitivity, so as to increase the number of correct fixation saccades and rereference the oculomotor system. There could be improvement in ocular motor control and in “searching capacity.” Furthermore, learning to use eccentric fixation could be a mechanism contributing to amelioration. Another suggestion is an increase in the discriminating capacities of both the retina and the visual cortex and associated areas.

Cerebral plasticity is likely to play an important role. Neurons are thus able to respond to weaker stimuli compared to when responding to no attention. Alpeter demonstrated that attention also increases the coherence between neurons responding to the same stimulus [18].

The biofeedback effect is related to the brain's ability to perceive an efficient PRL for visual tasks. The audio feedback can help the brain to fix the final PRL by increasing the attentional modulation. The structural stimulus involved visual receptive fields highly sensitive to medium spatial frequencies, and it is more effective than simple unstructured light stimulation as used in IBIS (improved biofeedback integrated system) device [4, 23].

Andrade et al. have shown that patients are usually unaware of their scotoma because, whenever the retina is damaged by a local lesion (induced scotoma), the cortical neurons driven by stimuli originating in this region do not remain inactive but become selective to stimuli originating in other parts of the retina. This process occurs in two distinct steps, each with its own time scale: (i) a fast redistribution of receptive fields (RFs) in the area of the lesion and (ii) a long-term reorganization that leads to the final RF configuration. Although the mechanisms underlying the gradual rearrangement are becoming clearer, the first step remains obscure. Cortical neurons located in the retinotopic position corresponding to the scotoma receive some degree of activity from the unimpaired neurons in the area surrounding the lesion [27].

Cortical plasticity allows the brain to adapt to background modifications or to nervous system damage. It also underlies learning and attention processes. Cortical changes occurring after focal visual differentiation modify visual perception by filling in visual field defects with information from the area surrounding the scotoma. This modification causes affected subjects to ignore or underestimate their defects. With visual field defects, cortical plasticity also causes distortion in spatial perception. These effects cause delay in the identification of visual field defects, and hence the initiation of therapy, while also affecting the results of some procedures to test the visual field [28]. Microperimetric biofeedback trains the neurotransmission chain to increase intercellular neurotransmitters and to restore neurobrain connections faster than in normal conditions.

In our study we reported a significant improvement in both visual acuity and microperimetric parameters. These findings suggest using microperimetric biofeedback as a rehabilitative strategy after retinal detachment surgery, considering also that it is a feasible and safe treatment. Unfortunately, in our study the follow-up time is limited to 18 weeks, and it may be useful to follow the patients for a long-term period.

## Figures and Tables

**Table 1:** Retinal sensitivity and bivariate contour ellipse area (BCEA) obtained in the two groups.

	Baseline	After BF
Retinal sensitivity, dB		
Group A	9.84 ± 2.03	15.42 ± 3.76
Group B	9.71 ± 2.21	10.2 ± 3.36
*P* value	0.83	<0.001
LogBCEA, deg^2^		
Group A	0.48 ± 0.17	0.04 ± 0.03
Group B	0.49 ± 0.2	0.40 ± 0.31
*P* value	0.87	<0.001

BF: biofeedback; dB: decibel; logBCEA: logarithmic transformation of bivariate contour ellipse area; all data are expressed as mean ± standard deviation; *P* value is calculated between groups by unpaired *t*-test.
